# Directional mass transport in an atmospheric pressure surface barrier discharge

**DOI:** 10.1038/s41598-017-14117-1

**Published:** 2017-10-25

**Authors:** A. Dickenson, Y. Morabit, M. I. Hasan, J. L. Walsh

**Affiliations:** Centre for Plasma Microbiology, Department of Electrical Engineering and Electronics, University of Liverpool, Liverpool, L69 3GJ United Kingdom

## Abstract

In an atmospheric pressure surface barrier discharge the inherent physical separation between the plasma generation region and downstream point of application reduces the flux of reactive chemical species reaching the sample, potentially limiting application efficacy. This contribution explores the impact of manipulating the phase angle of the applied voltage to exert a level of control over the electrohydrodynamic forces generated by the plasma. As these forces produce a convective flow which is the primary mechanism of species transport, the technique facilitates the targeted delivery of reactive species to a downstream point without compromising the underpinning species generation mechanisms. Particle Imaging Velocimetry measurements are used to demonstrate that a phase shift between sinusoidal voltages applied to adjacent electrodes in a surface barrier discharge results in a significant deviation in the direction of the plasma induced gas flow. Using a two-dimensional numerical air plasma model, it is shown that the phase shift impacts the spatial distribution of the deposited charge on the dielectric surface between the adjacent electrodes. The modified surface charge distribution reduces the propagation length of the discharge ignited on the lagging electrode, causing an imbalance in the generated forces and consequently a variation in the direction of the resulting gas flow.

## Introduction

Plasmas generated far from thermodynamic equilibrium in humid air at atmospheric pressure produce large fluxes of Reactive Oxygen and Nitrogen Species (RONS) including O, OH, O_3_, H_2_O_2_, NO and O_2_^−^^1–3^, These unique and highly reactive chemistries, accessible under ambient conditions, have opened the gateway to a wealth of new application domains spanning the fields of high-value manufacturing, environmental remediation and healthcare technologies^4–7^.

Of all the plasma systems currently under investigation to generate and sustain a stable non-thermal discharge under ambient air conditions, the Surface Barrier Discharge (SBD) configuration is one of the most commonly used due to its prevalence in small scale commercial ozone generation devices^5^. The SBD configuration offers numerous advantages over alternative designs due to its unique construction, typically comprising of metallic electrodes adhered to opposing sides of a thin dielectric material. On application of a sufficiently high voltage, plasma forms around the electrode edges and propagates across the dielectric surface. Typical advantages of the configuration include ease of scalability, with such discharges routinely used on an industrial scale, and exceptional energy efficiency^5,8^. A drawback of the SBD configuration is that the highly reactive neutral and charged species produced are essentially confined to the active (visible) discharge region, which is located close to the dielectric surface («mm). For many established applications, such as ozone generation, this does not present a problem as it is the long-lived species that are the primary application enablers. Conversely, in many emerging applications such as those in the healthcare sector, it is the short-lived RONS that are primarily responsible for the observed effects; examples include the use of plasma generated NO to stimulate wound healing and the role of O and OH in plasma mediated biofilm decontamination^9–12^.

In healthcare related applications of the SBD, a spatial separation of » 1 mm is commonly employed between the active plasma region and the sample to be treated; this removes the possibility that intense plasma filaments could interact directly with a delicate substrate (*e*.*g*. living tissues) and limits the potential for cross-contamination of the sample and electrodes. In such a scenario, it is extremely unlikely that charged species or highly reactive neutrals will reach the downstream sample, having a negative impact on application efficacy^13^. To overcome these challenges, several studies have posited that plasma induced electrohydrodynamic (EHD) forces could be manipulated to induce a convective gas flow through the active discharge region, thus transporting the species generated downstream and enhancing mass-transport^8,13,14^. Indeed, the previous computational studies of Hasan *et al*. have demonstrated that such convective flows could enable the transport of relatively short-lived species, such as OH and NO, to considerable distances downstream of the active discharge region^13^. Furthermore, it has been demonstrated that the introduction of a convective flow impacts the generation and loss rates of species in the downstream gas region, resulting in enhanced production of key RONS^3,13^.

In this contribution, a phase shift between the voltage waveforms applied to two adjacent electrodes in a surface barrier discharge is applied to exert a level of control over the direction of the EHD force, facilitating the targeted delivery of plasma generated reactive species to a given a given downstream spatial location. Previous studies exploring the use of SBD devices for aerodynamic flow control applications have reported that the direction of the induced flow can be varied by manipulating the amplitude of the applied voltage or by pulse modulating the voltage applied to adjacent electrodes^15–17^. While these approaches are effective in steering the induced flow, and therefore meeting the requirements of their intended application, they both rely on compromising plasma formation on one of the electrodes and are therefore not ideal for use when the density and nature of the plasma generated RONS are the key application drivers.

## Results

**Figure 1:**
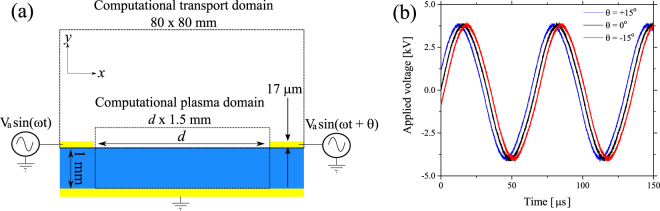
(**a**) Surface barrier discharge electrode configuration, showing the experimental configuration with the computational model domain superimposed, all key dimensions in the model were chosen to match the experimental setup. For the computational model, the highlighted plasma domain is the region where the equations describing the plasma are solved and the transport domain is where the flow equations are solved, and (**b**) phase shifted sinusoidal voltage waveforms applied to the right-hand electrode. The model was solved for d = 5 mm case only.

Two high voltage sinusoidal power sources were used to energise adjacent electrodes in the SBD configuration shown in Fig. 1(a). A quartz sheet of 1 mm thickness was used as the dielectric material and a single grounded electrode was positioned beneath the quartz to form a counter electrode for both powered electrodes. It is well known that adjacent strip electrodes produce an EHD generated flow in a direction perpendicular to the dielectric material^18^. In both experimental and numerical investigations, one electrode was energised with a sinusoidal voltage having a phase angle of 0°, a second sinusoidal waveform was applied to the other electrode with a variable phase angle between +/−60°. Figure 1(b) shows the high-voltage waveforms applied to the powered electrodes with a phase difference of +/−15°.

**Figure 2:**
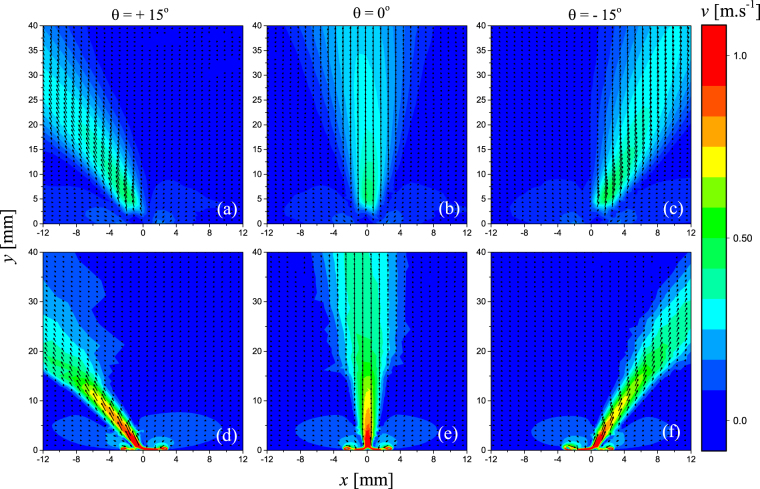
(**a**–**c**) Measured velocity vector maps showing impact of a +15°, 0° and −15° phase difference between applied voltages, respectively. (**d**–**f**) Calculated velocity vector maps showing impact of +15°, 0° and −15° phase difference between applied voltages, respectively. All cases correspond to an electrode separation distance of 5 mm.

Particle Imaging Velocimetry (PIV) was used to quantify the resulting gas flow velocity from the arrangement shown in Fig. 1. Critically, in the context of an SBD, the flow through the discharge region is the primary mechanism for the mass transport of medium- and long-lived plasma generated RONS^13^. Figure 2(a–c) shows the measured velocity profile produced by the discharge generated from two adjacent electrodes placed 5 mm apart and energised with a high-voltage sinusoidal waveforms employing a phase difference of +15°, 0° and −15°, respectively. Clearly, the introduction of a phase difference between the voltages applied to the individual electrodes results in a shift in the direction of the downstream convective flow generated by the plasma. To assist in identifying the underpinning mechanisms responsible for the observed variation in flow direction, a 2D air plasma model was developed. Figure 2(d–f) shows the calculated velocity profiles obtained by applying phase shifts of +15°, 0° and −15° to one electrode, under identical conditions to those used in the experiment. As can be seen, the simulation data closely matches that measured experimentally in the region downstream of the discharge. In the area around the discharge, interference by light emission from the plasma, reflected laser light from the surface and the limitations of the PIV technique to simultaneously capture low and high velocities reduces the accuracy, thus explaining the discrepancy between the numerical and experimental data.

**Figure 3:**
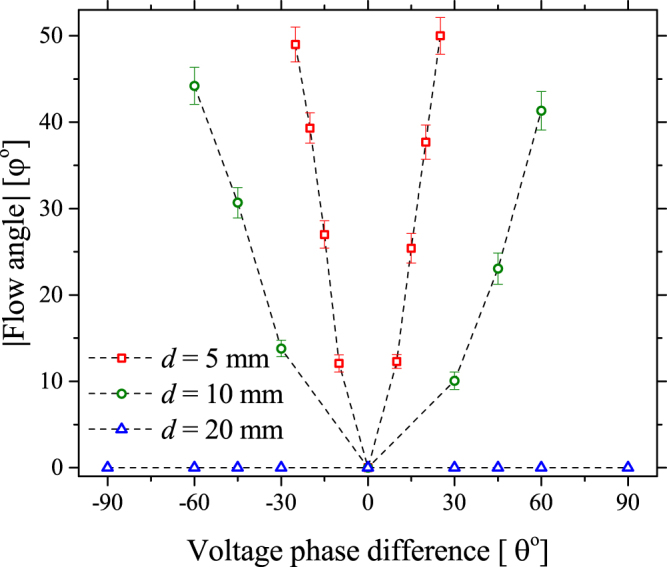
Measured deviation of the electrohydrodynamic flow direction from the perpendicular as a function of applied voltage phase difference and electrode separation distance at a constant applied voltage amplitude and frequency of 4 kV and 15 kHz, respectively.

The results presented in Fig. 2 were obtained using a spatial separation between the two electrodes of 5 mm (*d* = 5 mm). The spatial separation between the electrodes was found to be a critical parameter influencing the extent to which the voltage phase difference caused a shift in the direction of the generated gas flow. Figure 3 shows the angle of the generated flow (φ) obtained from PIV measurements as a function of the applied voltage phase difference (θ) and electrode separation distance (*d*). From the presented data, it is clear that an increased electrode separation distance yields a less pronounced variation in the angle of the induced flow. At large electrode separations, considered to be *d* => 20 mm in this investigation, the generated flow shows no dependence on the voltage phase difference.

**Figure 4:**
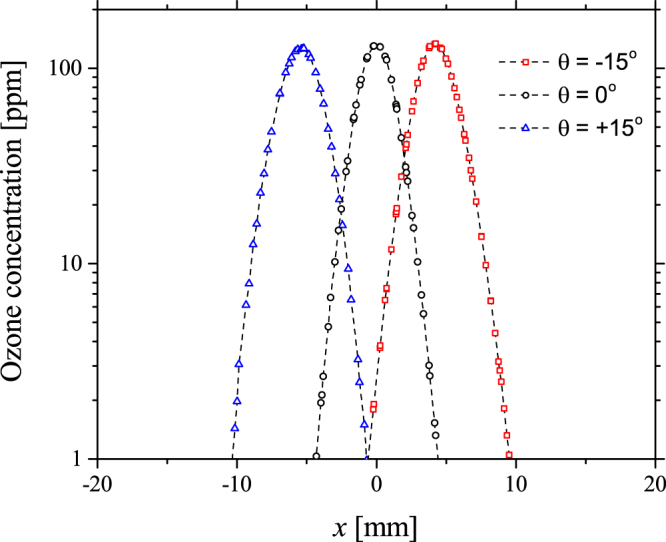
Calculated ozone density 10 mm from the active discharge region (*y* = 10 mm), for applied voltage phase differences of +15°, 0° and −15°, for an electrode separation of 5 mm. The x-axis is centred on the midpoint between the two electrodes.

From the results presented in Figs 2 and 3 it is clear that the introduction of a voltage phase difference between the two adjacent electrodes can be used to influence the direction of the induced flow. While it is important from an application perspective to be able to electrically control the direction of species transport and delivery, it is also vital that the method used to achieve this directionality does not impede the production of RONS within the discharge region. To confirm this, the numerical model was employed to calculate the density distribution of ozone at a point 10 mm above the active discharge region for applied phase differences of +15°, 0° and −15°, shown in Fig. 4. Regardless of the voltage phase difference, the generated ozone density is seen to remain constant providing a strong indication that the underpinning generation mechanisms are not compromised. Furthermore, the spatial position at which the peak ozone density occurs corresponds directly with the highest velocity region shown in Fig. 2, confirming that the EHD generated flow is indeed the main mechanism of species transport.

## Discussion

While the results presented in Figs 2–4 fully demonstrate the utility of the approach, the underpinning mechanisms behind the phenomenon are yet to be explained and likely differ significantly from other studies examining directional control of flow for aerodynamic applications. In previous studies, efforts have been directed toward reducing the flow generated from one electrode and thus enabling the flow from the other electrode to dominate, yielding a change in direction of the overall fluid flow. The simplest way to achieve this is to use a lower voltage on one electrode, which results in an EHD force field that is weaker on the lower voltage electrode causing the flow to tilt; alternatively, pulsing the voltage to the electrodes with a significant time delay (>ms) enables the flow from one electrode to fully develop before the other is ignited resulting in a shift in direction. Both of these approaches are likely to have a significant impact on the production of chemical species in the plasma and are therefore not well suited for use in applications where the chemical species are the primary application driver. In this study, a phase shift is employed between the voltage waveforms applied to adjacent electrodes. As the operating frequency employed in this study is a constant 15 kHz, a phase difference of +/−15° represents a time delay of +/−2.78 µs between the two waveforms, a value that is several orders of magnitude lower than those used in previous studies^17^. As the fluid flow is known to develop on a millisecond timescale in a dielectric barrier discharge it is surprising that such small time delays have a significant impact on the direction of the fluid flow.

**Figure 5:**
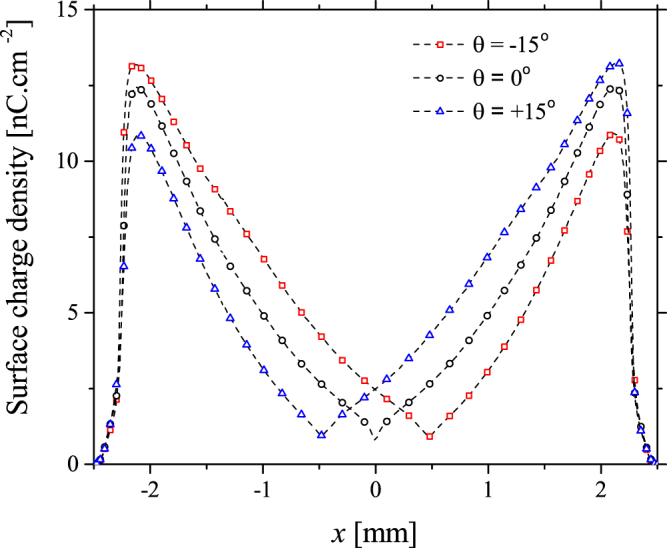
Calculated surface charge density for applied voltage phase differences of +15°, 0° and −15° for an electrode separation of 5 mm, with the electrode edges situated at −2.5 mm and +2.5 mm, respectively.

Given that the change in flow direction is a function of the spatial separation between the adjacent electrodes, it is reasonable to assume that the phenomenon occurs due to a mutual interaction between the discharges generated on each electrode edge. As the two discharges are placed closer together, their interaction becomes increasingly intense. In an SBD, streamers form during the positive half cycle of the applied voltage waveform, these are ignited at the edge of the electrode and propagate rapidly (<50 ns) across the dielectric surface, resulting in charge deposition on the dielectric material. Streamer propagation continues until the electric field at the streamer tip becomes insufficient to initiate new electron avalanches. Upon termination, the quasi-neutral region behind the streamer tip gradually decays through a process of recombination and further charge deposition on the dielectric surface. During the negative half cycle, a glow-like discharge is ignited under the influence of the deposited surface charge remaining from the positive half cycle. When a second, adjacent electrode is placed in close proximity, discharges form on both electrodes and the possibility for mutual interaction arises. To unravel the nature of this interaction, the numerical model was employed to capture the dynamics of the adjacent phase shifted discharges. The results from the model indicated that the discharges in the positive cycle are coupled through the surface charge they deposit within the space between the electrodes. The deposited surface charge density profiles are shown in Fig. 5 for the +15°, 0° and −15° phase differences.

The surface charge density profiles presented in Fig. 5 demonstrate that a phase difference between the applied voltage waveforms causes a distortion in the deposition of charge on the dielectric surface. Taking the −15° phase difference case as an example, during the positive cycle a streamer originating from the electrode positioned at −2.5 mm is the first to be ignited due to the phase difference and subsequently propagates towards the adjacent electrode depositing charge on the dielectric surface. A short time later, a second streamer is ignited from the adjacent electrode situated at +2.5 mm and begins to propagate; however, the positive charge deposited by the first streamer covers more than half of the dielectric surface between the electrodes. The presence of this positive charge on the dielectric surface impedes the propagation length of the second streamer. As a result, the minimum surface charge density point is closer to the electrode situated at +2.5 mm. Essentially, propagation is terminated at *x* = 0.5 mm for both streamers, meaning the length of the streamer originating from the electrode at −2.5 mm is 3 mm and the streamer originating from the electrode at +2.5 mm is 2 mm. When a phase difference of +15° is applied, the same process occurs with the first streamer originating from the electrode edge at +2.5 mm and the minimum surface charge point is at *x* = −0.5 mm, closer to the −2.5 mm electrode. In the case when no phase difference is applied, both streamers ignite simultaneously, demonstrated by the location of the minimum surface charge point being exactly in the middle between the two electrodes.

**Figure 6:**
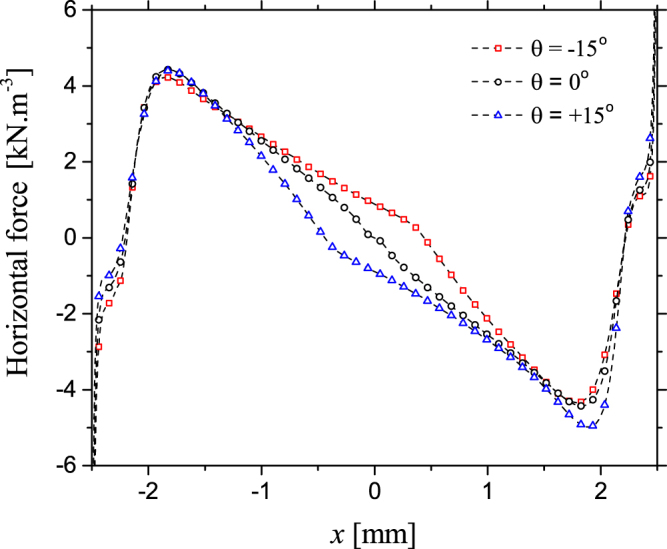
Calculated time-averaged horizontal force density generated by the plasma for applied voltage phase differences of +15°, 0° and −15°, shown along a cut line at y = 20 μm.

In an SBD, the EHD force is generated by the movement of charged species drifting in the electric field, resulting in momentum transfer to neutral species through repeated collisions. In the positive half cycle, this force is exerted as the streamer tip propagates away from the electrode. Given that the localised electric field in the streamer is determined by the voltage difference between the streamer and the deposited surface charge, it is to be expected that any distortion in the surface charge profile would subsequently impact upon the force generated by the propagating streamer^19^. In the negative half cycle, a discharge ignites between the deposited charge on the surface and the electrode, given that the surface charge density is altered by the applied phase shift, the EHD force exerted in the negative cycle is also modified. Figure 6 shows the time-averaged force density exerted by the discharge generated from adjacent electrodes for phase differences of −15°, 0° and +15° in a single period, along a line just above the electrodes (y = 20 μm). Being a time-averaged force density it includes the contribution of discharges in both the positive and the negative half cycles of the applied voltage waveform.

From Fig. 6 it can be seen that the introduction of a phase difference results in a shift of the equilibrium position of the horizontal force density (the point where the force is zero), indicating that the force exerted by the leading discharge acts over a larger volume than the lagging discharge. Interestingly, the location of the equilibrium point coincides with the minimum surface charge points shown in Fig. 5. The implication of a shift in the equilibrium position is that a net horizontal force is exerted on the flow by the force acting on a larger volume. Consequently, a net momentum is transferred to the gas between the electrodes in the x-direction, causing the direction of the resulting gas flow to diverge from the expected perpendicular direction. In the case of a −15° phase difference, the equilibrium position of the force is shifted towards the electrode situated at +2.5 mm, thus the resulting gas flow is observed to bend towards the right-hand side, as seen in Fig. 2(c),(g). Conversely, a +15° phase difference causes a shift in the equilibrium position of the force to the left, thus causing the resulting gas flow to bend to the left-hand side, as seen in Fig. 2(a) and (d). When no phase difference is applied, the equilibrium position of the force is in the centre of the dielectric surface, and no net momentum is transferred to the gas between the electrodes in the x-direction, resulting in gas flow that is directed away from the dielectric surface in a perpendicular direction, as seen in Fig. 2(b) and (e). Critically, the difference between the steering method proposed here and alternate approaches is that the flow is steered by manipulating the volume over which the EHD force is exerted, instead of manipulating the amplitude of the EHD force itself. As the electrodes are moved further apart, the localised electric field in the region around each electrode becomes less distorted by the presence of the discharge igniting from the adjacent electrode. In this situation, the force exerted by each discharge remains constant and no force imbalance occurs regardless of the applied voltage phase difference, explaining the results presented for *d* = 20 mm in Fig. 3.

## Summary

In this contribution, a technique is reported that enables the direction of reactive species transport in a SBD to be manipulated without compromising the generation efficacy of the reactive species. By introducing a phase difference between the voltages applied to adjacent electrodes in an SBD an imbalance in the EHD forces generated by the discharges occurs, yielding a change in the direction of the EHD induced gas flow. Voltage phase differences between +/−60° were investigated and shown to give rise to shifts in the gas flow direction of +/−50° from the conventional perpendicular direction. A 2D numerical air plasma model was used to investigate the underpinning physical processes giving rise to the observed phenomenon. Using the model it was determined that an interaction occurs between the two adjacent discharges as a result of a distortion in the charge deposition profile in the region between the two electrodes. The profile of charge distribution in this region influences the localised electric field which in turn impacts the EHD forces generated by each plasma. Through the introduction of a phase difference between the applied voltage waveforms, it was established that one discharge was able to propagate longer and exert force in its direction of propagation over a larger distance compared to that exerted by the discharge originating from the opposite electrode, resulting in a shift in flow direction.

As the generation of reactive plasma species is not compromised by the introduction of a phase difference, this technique can be safely used to facilitate the targeted delivery of plasma generated RONS to a given downstream location without compromising generation efficacy. Essentially, the technique could be used to enable large area samples to be treated using a smaller and potentially non-uniform SBD electrode. Alternatively, the technique could be employed to deliver differing compositions of RONS to targeted spatial location on a downstream sample, facilitating patterning etc.

## Methods

### Plasma system

**Figure 7:**
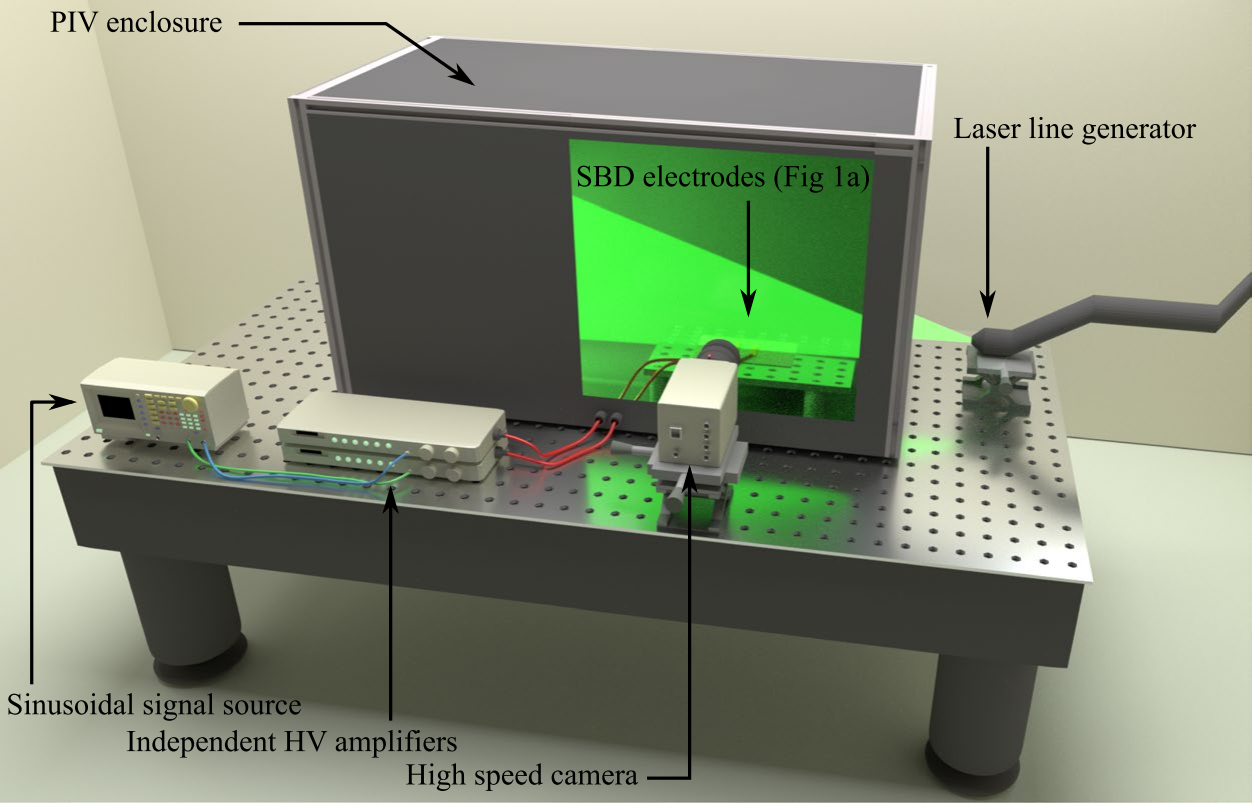
Depiction of experimental arrangement used to undertake Particle Imagining Velocimetry measurements.

The low temperature plasma system used in this investigation consisted of two home-made high-voltage power amplifiers driven by a single low-voltage sinusoidal signal generator (Tektronix AFG3101C). The signal generator provided two sinusoidal outputs at a constant frequency of 15 kHz and the functionality to introduce a user-programmable phase shift between the two generated waveforms. The phase shifted waveforms were used as inputs to the two high-voltage amplifiers and a gain was set to give a 4 kV amplitude output. The high-voltage waveforms were connected directly to the electrodes shown in Fig. 7 and schematically in Fig. 1(a). A calibration procedure was employed to ensure that the voltages applied to each electrode were a constant 4 kV amplitude throughout all experiments.

### Particle imaging velocimetry

The SBD electrode was placed within a chamber measuring 1000 mm wide, 1000 mm tall and 1500 mm long to contain the seeding particles, shown Fig. 7. Global measurements of the velocity field were taken along the centreline of the SBD device with a time-resolved particle image velocimetry (PIV) system from TSI Incorporated. The PIV system employed a double pulse Nd:YLF laser operating with a pulse duration of 100 ns at 527 nm. The timing between two adjacent laser pulses is the critical factor and was set to ~130 µs, a value chosen to give the time-averaged velocity over two applied voltage cycles. A Phantom Micro 340 high-speed camera was used to capture a single laser pulse in each frame. A total of 800 frames, enough data to create 400 vector maps were captured in 1 second. To produce the velocity profiles shown in Fig. 2, all individual vector maps were averaged to produce a single time-averaged map of the steady state operating conditions of the discharge. To ensure steady state conditions, the discharge was ignited 1 second prior to capturing PIV data. All measurements were conducted inside a closed chamber to ensure the EHD flow was not influenced by any external draughts. Figure 7 also shows the position of the laser sheet within the PIV enclosure in relation to the SBD device. Oil with a nominal size of 1 µm was used to seed the air within the enclosure. The Stokes numbers of the seeding particles used throughout the study was < 0.1, thus ensuring that the seeding particles followed the fluid flow closely with tracing errors being < 1% ^20,21^. The velocity vectors were computed on a square grid with spatial resolution of 56 µm using a recursive cross-correlation technique.

### Computational model

The model used in this study consisted of two 2D sub-models (referred to as models, hereafter), a plasma model and a chemical transport model. The plasma model was only solved in the plasma domain shown in Fig. 1(a), it was a plasma fluid model that solved a system of conservation equations given by Equation (1). Assuming air consists of 79% N_2_ and 21% O_2_, Equation (1) was solved for the densities of 6 species which are electrons, ${{\rm{N}}}_{{\rm{2}}}^{+}$, ${{\rm{O}}}_{{\rm{2}}}^{+}$, ${{\rm{O}}}_{{\rm{2}}}^{-}$, O, and O_3_, in addition to the background O_2_ and N_2_.

1 $$\frac{\partial {n}_{k}}{\partial t}+\nabla \cdot {\mathop{{\rm{\Gamma }}}\limits^{\rightharpoonup }}_{k}={\sum }_{l}{k}_{lj}{n}_{l}{n}_{j}$$

2 $${\mathop{{\rm{\Gamma }}}\limits^{\rightharpoonup }}_{k}=-{\mu }_{k}{n}_{k}\nabla V-{D}_{k}\nabla {n}_{k}$$

In Equations (1) and (2), *n*_*k*_ is the density of the k^th^ species, *Γ*_*k*_ is the flux of the k^th^ species, *k*_*lj*_ is the two-body reaction coefficient, μ_*k*_ is the mobility of the k^th^ species, *D*_*k*_ is the diffusion coefficient of the k^th^ species, and *V* is the electric potential. A list of the reactions included in the model is given in Table 1.

**Table 1:** List of chemical reactions included in the model

Rxn No	Reaction formula	Reaction coefficient	Energy cost (eV)	Ref.
R1	e + N_2_ → e + N_2_	f(*ε*_*avg*_)^b^	0.3(T_e_ − T_g_)	22
R2	e + O_2_ → e + O_2_	f(*ε*_*avg*_)^b^	0.26(T_e_ − T_g_)	22
R3	e + N_2_ → 2e + N_2_^+^	1 × 10^−16^ *ε*_*avg*_^1.9^ exp(−14.6/*ε*_*avg*_)	15.58	1
R4	e + O_2_ → 2e + O_2_^+^	9.54 × 10^−12^ *ε*_*avg*_^−1.05^ exp(−55.6/*ε*_*avg*_)	12.07	1
R5	e + O_2_ → O_2_^−^	9.72 × 10^−15^ *ε*_*avg*_^−1.62^ exp(−14.2/*ε*_*avg*_) *ε*_avg_> 1.13 2.78 × 10^−20^ *ε*_avg_ < 1.13		1
R6	e + N_2_ + O_2_ → N_2_ + O_2_^−^	1.1 × 10^−43^ (T_g_/T_e_)^2^ exp(−70/T_g_) exp(1500(T_e_ − T_g_)/(T_e_T_g_))		1
R7	e + 2 O_2_ → O_2_ + O_2_^−^	1.4 × 10^−41^ (T_g_/T_e_) exp(−600/T_g_) exp(700(T_e_ − T_g_)/(T_e_T_g_))		1
R8	M + e + N_2_^+^ → M + N_2_	3.12 × 10^−35^/T_e_		1
R9	N_2_ + O_2_^−^ → e + O_2_ + N_2_	1.9 × 10^−18^(T_g_/300)^0.5^ exp(−4990/T_g_)		1
R10	O_2_ + O_2_^−^ → e + O_2_ + O_2_	2.7 × 10^−16^(T_g_/300)^0.5^ exp(−5590/T_g_)		1
R11	O_2_ + N_2_^+^ → O_2_^+^ + N_2_	5 × 10^−17^		1
R12	O_2_^−^ + N_2_^+^ → O_2_ + N_2_	2 × 10^−13^(300/T_g_)^0.5^		1
R13	O_2_^−^ + O_2_^+^ → 2 O_2_	2 × 10^−13^(300/T_g_)^0.5^		1
R14	e + O_2_ → 2O + e	2.03 × 10^−14^ *ε*_*avg*_^−0.1^ exp(−8.47/*ε*_*avg*_)	6.12	1
R15	M + 2O → M + O_2_	3.2 × 10^−47^exp(900/T_g_)		1
R16	M + O + O_2_ → M + O_3_	3.4 × 10^−46^ (300/T_g_)^1.2^		1
R17	O + O_3_ → 2O_2_	8 × 10^−18^ exp(−2060/T_g_)		1
R18	M + O_3_ → M + O + O_2_	3.92 × 10^−16^ exp(−11400/T_g_)		1

^a^ *ε*_*avg*_: Mean electron energy (eV), ${T}_{e}=2{q}_{e}{\varepsilon }_{avg}/(3{k}_{B})$, where k_B_ is Boltzmann constant, T_g_ is the gas temperature (K)

^b^f(*ε*_*avg*_) indicates a rate coefficient that is calculated from BOSIG+^22^.

In addition, the plasma model solved the electron energy conservation equation given by Equation (3).

3 $$\frac{\partial {n}_{en}}{\partial t}+\nabla \cdot {\mathop{{\rm{\Gamma }}}\limits^{\rightharpoonup }}_{en}={\sum }_{l}{\theta }_{l}{k}_{lj}{n}_{l}{n}_{j}-\nabla V\cdot {\mathop{{\rm{\Gamma }}}\limits^{\rightharpoonup }}_{e}$$

4 $${\mathop{{\rm{\Gamma }}}\limits^{\rightharpoonup }}_{en}=-{\mu }_{en}{n}_{en}\nabla V-{D}_{en}\nabla {n}_{en}$$

In Equations (3) and (4), *n*_*en*_ is the electron energy density, *θ*_*l*_ is the electron energy cost of the l^th^ reaction, *Γ*_*e*_ is the electron flux, *μ*_*en*_ and *D*_*en*_ are the electron energy mobility and diffusion coefficients, calculated using BOLSIG +, respectively^22^. The system of conservation equations were coupled with the Poisson equation, which was solved for the electric potential as given by Equation (5).

5 $${\nabla }^{2}V=\frac{-{q}_{e}}{{\varepsilon }_{0}}({n}_{N2}^{+}+{n}_{O2}^{+}-{n}_{O2}^{-}-{n}_{e})$$

In Equation (5), *q*_*e*_ is the elementary charge, *ε*_0_ is the free space permittivity. To follow the dielectric surface charging process, the surface charge continuity equation, given by Equation (6), was solved on the dielectric surface, shown as the interface between the air domain and the dielectric domain in Fig. 1(a).

6 $$\frac{\partial {\rho }_{s}}{\partial t}={q}_{e}\hat{n}\cdot ({\mathop{{\rm{\Gamma }}}\limits^{\rightharpoonup }}_{{N}_{2}^{+}}+{\mathop{{\rm{\Gamma }}}\limits^{\rightharpoonup }}_{{O}_{2}^{+}}-{\mathop{{\rm{\Gamma }}}\limits^{\rightharpoonup }}_{{O}_{2}^{-}}-{\mathop{{\rm{\Gamma }}}\limits^{\rightharpoonup }}_{e})$$

In Equation (6), *n* is the normal unit vector on the dielectric surface, *Γ* represents the fluxes of the charged denoted species. The dielectric height was set to 1 mm, the distance between the electrodes was set to 5 mm, and the height of the electrodes was set to 17 µm. The reference voltage had a peak amplitude of 4 kV and a frequency of 15 kHz. The phase shifted waveforms were identical to that of the reference waveform except being shifted by −15°, 0°, and 15°, respectively. All parameters in the model were chosen to match those used in experiments. The temperature in the simulation was assumed to be 27 °C. The plasma model was solved for a single period of the non-phase shifted waveform, corresponding to a total solution time of 66.67 μs. As the model was solved, the instantaneous EHD forces, given by Equation (8), and the instantaneous generation rate of O_3_, given by Equation (9), were integrated in time.

7 $${\mathop{F}\limits^{\rightharpoonup }}_{EHD}=-{q}_{e}({n}_{N2}^{+}+{n}_{O2}^{+}-{n}_{O2}^{-}-{n}_{e})\nabla V$$

8 $${R}_{O3}={k}_{16}{n}_{M}{n}_{O}{n}_{O2}$$

In Equation (8), *k*_16_ is the rate coefficient for reaction 16 given in Table 1, *n*_*O*_ and *n*_*O2*_ are the densities of atomic oxygen and molecular oxygen respectively, *n*_*M*_ is the density of a third species. At the end of the period, the time integral of the instantaneous EHD force field and instantaneous O_3_ generation rate were then divided by the waveform period, giving the time-averaged EHD force field and O_3_ generate rate. Those time-averaged quantities were used as inputs to the chemical transport model. The chemical transport model was solved in the transport domain shown in Fig. 1(a), it solved the continuity equation of the gas mixture (air and O_3_), as given in Equation (9), the Navier-Stokes equation for the gas mixture, as given in Equation (10) and the mass conservation equation for O_3_ as given in Equation (11). The chemical transport model was solved for 1 second, which was enough to reach steady state conditions at approximately 150 ms.

9 $$\frac{\partial \rho }{\partial t}+\nabla \cdot (\rho \mathop{u}\limits^{\rightharpoonup })=0$$

10 $$\rho \frac{\partial \mathop{u}\limits^{\rightharpoonup }}{\partial t}+\rho (\mathop{u}\limits^{\rightharpoonup }\cdot \nabla )\mathop{u}\limits^{\rightharpoonup }=-\nabla P+\nabla \cdot [\eta (\nabla \mathop{u}\limits^{\rightharpoonup }+\nabla {\mathop{u}\limits^{\rightharpoonup }}^{T})-\frac{2}{3}\eta (\nabla \cdot \mathop{u}\limits^{\rightharpoonup })I]+{\bar{F}}_{EHD}$$

11 $$\frac{\partial {n}_{O3}}{\partial t}+\nabla \cdot {\mathop{{\rm{\Gamma }}}\limits^{\rightharpoonup }}_{O3}={\bar{R}}_{O3}-{k}_{17}{n}_{O}{n}_{O3}-{k}_{18}{n}_{M}{n}_{O3}$$

12 $${\mathop{{\rm{\Gamma }}}\limits^{\rightharpoonup }}_{O3}={n}_{O3}\mathop{u}\limits^{\rightharpoonup }-{D}_{O3}\nabla {n}_{O3}$$

In Equations (9)–(12), ρ is the gas mixture density, *u* is the mixture velocity field, *P* is the pressure, *η* is the viscosity of air, ${\bar{F}}_{EHD}$ is the time-averaged EHD force field calculated from the plasma model, and ${\bar{R}}_{O3}$ is the time-averaged generation rate of O_3_ calculated from the plasma model.

### Data Availability

Data used to construct the figures is made available at: http://datacat.liv.ac.uk/.
